# Parental beliefs about positive affect and parental depressive symptoms predicting parents' positive emotion socialisation in India

**DOI:** 10.1002/ijop.12848

**Published:** 2022-05-04

**Authors:** McKenna Freeman, Anuradha Sathiyaseelan, Aaron Luebbe, Vaishali Raval

**Affiliations:** ^1^ Miami University Oxford Ohio USA; ^2^ Christ University Bengaluru India

**Keywords:** Positive affect, Parental emotion socialisation, Parental depression, India

## Abstract

Emerging literature examines implications of parental socialisation of positive affect (PA) for children's socio‐emotional functioning, though little is known about predictors of parental PA socialisation behaviours in diverse families around the world. Based on the literature that suggests that parental cognitions (Okagaki & Bingham, 2005) and their own mood state contribute to their parenting (Dix & Meunier, 2009), we examined two parent‐related factors (parental beliefs regarding PA and depressive symptoms) as predictors of parental responses to their adolescents' PA in an urban middle‐class sample of mothers and fathers from India (*N* = 267; 40.4% mothers). Parents completed measures of their PA‐related beliefs, depressive symptomatology, and their responses to adolescents' PA at two‐time points, 5 months apart. Parental PA‐related beliefs showed low stability and depressive symptoms showed moderate stability across time. There were concurrent bivariate associations between parental PA‐related beliefs and their socialisation behaviours, though these relations did not hold in multivariate path analyses across time. Parental depressive symptoms at T1 inversely predicted family savouring at T2 and positively predicted dampening at T2. These findings provide the first line of evidence indicating that parental cognitions and their own mood contribute to their emotion‐related parenting behaviours in India.

Positive emotions play an important role in adaptive outcomes including self‐esteem, enhancing creativity, and lowering the risk for depression (Lyubomirsky et al., 2005). Emerging literature suggests that what parents teach their children about positive affect (PA) has implications for children's skills for regulating PA (Katz et al., 2014; Yi et al., 2016), which in turn, contributes to risk for depression (Yap et al., 2008). However, substantially fewer studies examine factors that contribute to parental socialisation of PA. Developmental researchers suggest that parental cognitions (Okagaki & Bingham, 2005) and their mood (Dix & Meunier, 2009) influence their behaviours. Within emotion socialisation literature, parents' beliefs about emotions have been shown to be cross‐sectionally related to their socialisation of negative emotions in a sample from the United States (Lozada et al., 2016). With respect to parental mood, depressive symptoms in particular have been shown to compromise parenting competence in samples from the USA (Dix & Meunier, 2009). However, this research has been conducted with predominantly White samples from Western countries. Little is known about predictors of parental emotion socialisation in culturally diverse groups around the world. Furthermore, even with White American samples, studies that longitudinally examine factors contributing specifically to parents' PA socialisation have been rare. The purpose of the current study was to examine whether two parent‐related factors (parents' PA‐related beliefs and depressive symptomatology) were associated with their responses to their adolescents' PA within and across time in a sample of mothers and fathers from India.

## Parental socialisation of PA

Emotion socialisation occurs through parents' direct responses to their adolescents' emotions, discussions of emotions and parental modelling of their own emotional experiences (Eisenberg et al., 1998). Although a bulk of the research focuses on socialisation of negative emotions (Nelis et al., 2019), researchers have begun to examine how parents respond to adolescents' PA. Parents may respond to their children's PA by dampening (ignoring the child or telling them to calm down). Alternatively, parents may enhance PA (e.g., praising the child, expressing physical affection) (Katz et al., 2014; Luebbe, 2009; Yap et al., 2008) to help adolescents savour positive experiences by promoting the experience of PA for a longer duration of time, or discussing happy memories or future positive events (Luebbe, 2009).

Various adolescent outcomes have been associated with parental responses to adolescent PA. For example, lower parental enhancing responses to PA have been longitudinally associated with higher depressive symptoms in adolescents in Belgium (Nelis et al., 2019). On the contrary, cross‐sectionally, higher parental efforts to savour or enhance PA are associated with adolescents' own efforts to enhance PA and indirectly lower adolescent depression in the USA (Fredrick et al., 2019). Similarly, parental dampening responses to adolescent PA have been longitudinally associated with higher depressive symptoms amongst adolescents in India (Raval et al., 2018). Despite this knowledge of how parental responses influence adolescent outcomes, relatively little is known about why parents engage in specific behaviours in response to their adolescents' PA.

## Parents' emotion‐related beliefs predicting emotion socialisation behaviours

Parents' cognitions (e.g., beliefs, attitudes, expectations) influence their parenting behaviours (Okagaki & Bingham, 2005). Within this broader context, parents' beliefs and goals surrounding emotion also impact their emotion socialisation strategies and responses to their children (Dunsmore & Halberstadt, 1997; Eisenberg et al., 1998). Existing literature distinguishes parents' beliefs about PA as valuing PA and considering it to be useful, from beliefs that deem excessive PA as harmful or costly (Lozada et al., 2016; Parker et al., 2012).

Parents who believe that PA is valuable and prefer their children to have more frequent and lasting experiences of happiness, and they may respond to enhance such experiences. In contrast, parents who believe that too much PA can be harmful, also referred to as the “cost of positivity”, prefer their adolescents to modulate their PA experiences and may respond in ways that discourage PA. Indeed, two studies found that parental PA‐related beliefs were associated with their socialisation behaviours in the USA. For Lozada et al. (2016), the cross‐sectional findings were somewhat unexpected: parental belief that emotions are valuable was inversely related to parents' teaching of both positive and negative emotions. The researchers explained that parents who consider PA valuable may create opportunities for their children to experience PA but may not necessarily engage in direct conversation or teaching about PA. In another study, parental belief that PA is harmful or costly was uniquely associated with parents' dampening responses to adolescent PA (Nyquist & Luebbe, 2021). Both of these studies were cross‐sectional, and used different measures of parent responses (observational task, self‐report measure). It would be important to examine if these findings can be replicated in a sample from India, as well as to examine how parents' PA related beliefs contribute to their PA socialisation over time.

Parents' beliefs related to PA are largely dependent on the cultural and social context in which the family is located (Okagaki & Bingham, 2005). Beliefs, scripts and desires surrounding emotions differ across cultures, and scholars suggest that in Western cultures (e.g., USA, Canada, UK, the Netherlands), a common goal is to maximise PA and minimise negative affect (NA; Miyamoto & Ryff, 2011). Thus, parental beliefs that consider PA as valuable may fit with this cultural script. However, in Asian cultures (e.g., India, China) a common goal may be to achieve a balance between PA and NA (Miyamoto & Ryff, 2011), and thus, parental beliefs that consider too much PA as harmful and favour modulating PA may fit with this cultural script. These differing parental beliefs about the value of PA may impact their socialisation responses. Given cultural differences in emotion scripts, it is important to examine the association between parental beliefs and PA socialisation in samples from around the world. To address these gaps, in the current study, we examined the associations between parents' PA related‐beliefs and their responses to their adolescents' PA in a sample from India both within and across time. Replicating Nyquist and Luebbe's (2021) US‐based cross‐sectional finding, we expected that Indian parents' beliefs endorsing harmful effects of PA would be associated with their responses that discourage or dismiss adolescents' experiences of PA to help them modulate their affective experience. Given the unexpected finding of an inverse concurrent association between parents' beliefs endorsing value of PA and parents' PA socialisation in a US‐based sample (Lozada et al., 2016) and the cultural script in Asian cultures that focuses on a balance between PA and NA (rather than maximising PA), it was unclear what associations we can expect between parents' beliefs endorsing the value of PA and their responses to their adolescents' PA. Thus, we examined these associations in an exploratory manner. Extending the existing cross‐sectional work with US‐based samples, we examined whether parents' PA‐related beliefs predict their responses to adolescent PA over time in our sample from India.

## Parental depressive symptoms predicting parenting

When parents experience difficulties with their mental health, it would be no surprise that the quality of their parenting is affected. Parents' depressive symptoms have consistently been associated with less functional parenting (Dix & Meunier, 2009; Lovejoy et al., 2000). In particular, parents who experience depressive symptoms may have fewer positive responses to their children, more negative or disengaged responses, and inconsistent discipline, each of which have been associated with increased vulnerability to cognitive and emotional impairments of children (Dix & Meunier, 2009). Parental depressive symptoms have also been associated with negative child outcomes including internalising symptoms, negative attribution style, social difficulties and externalising problems in the USA (Downey & Coyne, 1990; Shelton & Harold, 2008). Despite substantial evidence suggesting that parental depression compromises parenting, there are two major gaps. First, a bulk of this research is conducted with Western samples, with relatively little evidence from other parts of the world. For example, parental depression has been associated with poorer family functioning in India (Jaiswal et al., 2015), although the relation between parental depression and parenting in India has not been tested. Second, relatively less research has examined the effects of parental depression specifically on how they respond to children's emotions (Lovejoy et al., 2000). In one study, parental depressive symptoms predicted parents' wish‐granting responses (i.e., parent giving in) to their toddlers' negative emotions in the USA (Premo & Kiel, 2016). In another, parental depressive symptoms uniquely predicted their dampening responses to their adolescents' PA in the USA (Nyquist & Luebbe, 2021). However, there is no published research to date that has investigated the association between parental depressive symptoms and PA socialisation outside of the USA.

Individuals with increased depressive symptoms tend to experience decreased PA (Clark & Watson, 1991), and thus, it would not be surprising to expect a parent experiencing depressive symptoms to minimise PA. Individuals with increased depressive symptoms report greater dampening of their own positive emotions (Werner‐Seidler et al., 2013) and may struggle to identify and respond to PA in others. In the current study, replicating Nyquist and Luebbe's (2021) US‐based cross‐sectional finding, we expected parents' depressive symptoms to be uniquely associated with higher dampening of adolescents' PA. In addition, we examined whether parents' depressive symptoms predict parental responses to adolescent PA over time. We chose a longitudinal design to help establish the direction of effect in the associations between parental PA‐related beliefs, depression and their socialisation behaviours.

## METHOD

### Participants

Participants were 281 parents (40.4% mothers, 53.9% fathers, 5.7% missing gender identity; *M*_age_ = 42.58 years) from an urban city in India with a child in grade (standards) 9 through 12 (*M*_age_ = 15.00 years, 84.7% girls). Of parents providing data, 96.4% were married. Parents endorsed family religion as 65.1% Hinduism, followed by 30.2% Christianity, 4.0% Islam and 0.7% Jainism. Number of total individuals in family homes ranged from 2 to 20 with three participants reporting between 12 and 20 individuals (*M* = 4.70, *SD* = 1.93). The number of children ranged from 1 to 6 (*M* = 2.00, *SD* = 0.64). A majority of participants had a monthly family income of 21,000–60,000 Rs. (51.5%) and parents' education ranged from less than standard 10 (2.1%), standard 10 complete (14.9%), standard 12 complete (19.5%), a few years of college (13.1%), a Bachelor's degree (32.6%), Master's degree (5.7%), to Doctoral/Professional degree (7.1%).

This project was approved by the Miami University Institution Review Board and all procedures were in accordance with the 1964 Helsinki Declaration and its later amendments or comparable ethical standards. Informed consent was obtained from all participants in this study.

### Procedure

At the beginning of the school year, study packets were sent home with all students in standards 9 through 12 (4160 families were reached). Interested parents were instructed to read and sign the consent form, complete questionnaires, and return packets to their child's school in a sealed envelope. Study materials were available in both English and Kannada, a local language, although all parents chose to complete questionnaires in English. High school students in India spend a substantial amount of time preparing for state examinations with parents providing close monitoring. To be responsive to participants' needs to attend to academics, T2 data were collected before students began their exam preparations, 5 months after T1. A total of 89.32% of parents from T1 completed the questionnaires for T2. Participants received educational supplies worth INR 150 (US $2.29).

### Measures

#### Demographics

Parents reported their gender, age, adolescent gender and age, number of individuals in the home, religion, and monthly income.

#### PA beliefs

Beliefs that PA is harmful were measured using the Parents' Beliefs about Children's Emotion Scale cost of positivity subscale which has been validated amongst North American samples (PBACE; 4 items; Halberstadt et al., 2013). Sample items include “When children are too happy, they can get out of control,” and “Too much joy can make it hard for a child to understand others.” Parents rate their agreement with each item on a scale from 1 (Strongly Disagree) to 6 (Strongly Agree). Cronbach's alpha revealed slightly low but adequate levels of reliability (α = 0.62 and 0.68, see Table 1).

**TABLE 1:** Descriptive statistics for the study variables (*N* = 281)

	1	2	3	4	5	6	7	8	9	10	11	12
1. Cost of Positivity (T1)	—											
2. Valuing Happiness (T1)	.29**	—										
3. Depression subscale (T1)	.16**	.05	—									
4. Family Savouring (T1)	.09	.24**	−.28**	—								
5. Enhancing (T1)	.06	.19*	−.10	.47**	—							
6. Dampening (T1)	.16*	.01	.32**	−.05	.09	—						
7. Cost of Positivity (T2)	.14*	.06	.10	.01	−.09	.06	—					
8. Valuing Happiness (T2)	.09	.28**	−.04	.20**	.05	.05	.27**	—				
9. Depression subscale (T2)	.07	−.06	.41**	−.21**	−.20**	.15*	.09	.00	—			
10. Family Savouring (T2)	.07	.17**	−.26**	.49**	.27**	.04	.11	.30**	−.21**	—		
11. Enhancing (T2)	−.03	.15*	−.05	.28**	.46**	−.02	.11	.23**	−.21**	.47**	—	
12. Dampening (T2)	.11	−.02	.41**	−.15*	−.10	.29**	.16*	.07	.52**	−.05	.06	**—**
Cronbach's alpha	0.62	0.79	0.81	0.92	0.90	0.72	0.68	0.78	0.86	0.91	0.89	0.75
Mean (SD)	3.92 (1.01)	4.80 (1.16)	6.02 (4.83)	3.80 (.81)	4.67 (1.18)	2.88 (1.20)	3.61 (1.03)	4.29 (1.15)	6.44 (5.25)	3.61 (.79)	4.36 (1.14)	2.93 (1.16)

*Note*: Correlations are pooled estimates across imputed data files (*N* = 40 imputations). Alphas, means, and standard deviations use original available data (*N*s range from 222 to 267). The total score for the depression subscale represents sum of all items on the scale for each participant; for all other scales, the total score represents mean item score.

Beliefs that PA is valuable were measured using the Valuing Happiness Scale which has been validated amongst North American samples (7 items; Mauss et al., 2011). Sample items include “Feeling happy is extremely important to me” and “To have a meaningful life, I need to feel happy most of the time.” Parents rate their agreement with each item on a scale from 1 (Strongly Disagree) to 7 (Strongly Agree). Cronbach's alpha revealed adequate levels of reliability (α = 0.79 and 0.78, see Table 1).

#### Parental depressive symptoms

Parental depressive symptoms were measured using the depression subscale of Depression Anxiety Stress Scales which has been validated amongst North American samples (DASS; Lovibond & Lovibond, 1993). The subscale includes seven items (e.g., I felt sad and depressed). Items are rated on a 4‐point scale (0 = did not apply to me at all to 4 = applied to me very much, or most of the time). The measure has demonstrated good reliability in samples from India (.95, Verma & Mishra, 2020) and the estimates in the current sample were adequate (α = 0.81 and 0.86, see Table 1).

#### PA socialisation

Parents completed a shortened version of the Responses to Adolescent Happy Affect Scale (RAHAS; Katz et al., 2014). The original RAHAS includes 13 vignettes regarding parental responses to adolescent expression of PA. In order to decrease burden, three of the 13 vignettes were chosen by our research team that were culturally relevant for school‐going adolescents in urban India. The scenarios included: “If your child is proud of him/herself for doing well on a test, I would … (9 possible responses, i.e., ‘excitedly tell other family members’, ‘tell him/her not to get so excited because one test doesn't make up one's whole grade’.),” “If your child comes home from school in a good mood, I would … (10 possible responses),” and “If your child is very excited about going out with friends, I would… (10 possible responses).” Parents rated the likelihood they would respond in each possible manner on a 1 (Very Unlikely) to 7 (Very Likely) scale. Academic achievement is highly valued in urban families in India, and thus, the theme of achievement is culturally relevant. Adolescents spend time with friends, including going out with them, making it a relevant context, and a generic context of being in a good mood also provides a relatable situation.

The subscales of the RAHAS include two overarching factors: enhancing responses and dampening responses (Katz et al., 2014). A factor analysis conducted at Time 1 revealed a poor overall fit for the two‐factor model (*χ* ^2^(376) = 928.87, *p* < .01; CFI = 0.69; RMSEA (90%CI) = 0.08 (0.07, 0.08); SRMR = 0.11). Four items failed to load onto the dampening factor. Two of these items assessed parental encouragement of children to work harder after receiving a good grade, and two items assessed parental probing for NA when their child expressed PA. After removal of these items, fit was acceptable (*χ* ^*2*^(268) = 455.32, *p* < .01; CFI = 0.87; RMSEA (90% CI) = 0.05(0.04, 0.06); SRMR = 0.08). This factor structure was reasonably replicated at with Time 2 data (χ^2^ (268) = 517.78, *p* < 0.01; CFI = 0.84; RMSEA (90%CI) = 0.06(0.05, 0.07); SRMR = 0.08). For analyses, mean item scores for dampening (8 items) and enhancing (17 items) at each time point, respectively, were used. Cronbach's alpha revealed adequate levels of reliability and ranged from 0.72 to 0.90 (see Table 1).

The Family Savouring Inventory (15 items; FSI; Luebbe, 2009) was also used to measure parental PA socialisation. The FSI measures parental beliefs of how their family responds to good things, such as a family member getting a good grade, winning an award, overcoming a difficult situation and so on. The scale includes questions such as: “When something good happens in my family we are able to enjoy it to the fullest,” “My family talks about positive events often,” and “We try hard to make good feelings last longer in my family.” Participants rate their responses on a scale of 1 (not at all true of my family) to 5 (very true of my family). Cronbach's alpha revealed high levels of reliability (α = 0.92 and 0.91, see Table 1).

## RESULTS

### Preliminary analyses

For primary variables, 13.20% of the data were missing. Variables with the most missingness included the RAHAS enhancement and dampening scales at T1 (21.0% and 17.8%, respectively; potentially given the structure of the measure, some participants may have been unclear on answering each item under each vignette leading to high levels of missingness for total scores). Little's MCAR test (*χ* ^*2*^ (1252) 1310.70, *p* = .121) suggested that data were consistent with pattern of missing completely at random. Missing data were handled with multiple imputations for preliminary analyses (*n* = 40 data sets using all primary variables and demographics in the algorithm) and full‐information maximum likelihood for primary aims.

Preliminary analyses included bivariate associations across all variables (see Table 1). Patterns of results were remarkably similar at T1 and T2. Findings of interest include that cost of positivity beliefs were concurrently associated with greater dampening responses at both T1 and T2, but unrelated to either savouring or enhancing. On the contrary, beliefs in the value of happiness were concurrently associated with savouring and enhancing responses at T1 and T2, respectively, but unrelated to dampening. Interestingly, cost of positivity and valuing happiness beliefs were positively associated with one another at both timepoints. Furthermore, at each time point, depressive symptoms were significantly inversely associated to family savouring but positively associated with dampening responses. At T1, depression was unrelated to enhancing, but these were modestly inversely related at T2. The current sample had a total mean score of 6.02 and 6.44 at T1 and T2, respectively, on the depression subscale. Total scores can range from 0 to 28, and a score of 9 or above indicates depressive symptoms in the clinical range (Lovibond & Lovibond, 1993). Although the current sample reported low depressive symptoms on average, 9.6% of participants at T1 and 10.7% at T2 were at or above the cut‐score of 9. Finally, for our dependent variables, there was moderate stability over time, with dampening demonstrating the most intraindividual change (though means were similar across time).

### Main analyses

A path analysis was conducted in Mplus v 7.4 (Muthen & Muthen, 1998–2018) to test our primary question of whether parental beliefs about PA and parental depressive symptoms predicted change in PA socialisation behaviours over 5 months (see Table 2). The path analysis allowed us to control for T1 values of socialisation behaviours while also testing all hypotheses in a single model, thereby controlling type I error inflation. Specifically, paths from parental cost of positivity beliefs (T1), parental beliefs in the value of PA (T1), and parental depressive symptoms (T1) to family savouring (T2), enhancing responses (T2) and dampening responses (T2) were estimated. T1 values of the dependent variables were included as predictors so that tests were measuring change in socialisation behaviours. Furthermore, older parents (*r =* .13, *p* = .047) and mothers (*r =* .21, *p* = .001) reported more family savouring at T2 and parents with more education reported less dampening at T2 (*r* = −.15, *p* = 017). As such, parent age, gender and education were included as covariates on relevant outcomes. No other demographic variables were associated with dependent variables in the model and thus were not considered further.

**TABLE 2:** Coefficients from path analysis predicting parental socialisation responses from PA beliefs and depressive symptoms

			95% CI			
	b	SE	LL	UL	*β*	p
	DV = T2 Family savouring (*R* ^*2*^ = .28)					
Parent age	−.01	.01	−.03	.01	−.07	.278
Parent gender	**.32**	**.10**	**.12**	**.53**	**.20**	**.002**
Family savouring (T1)	**.38**	**.06**	**.27**	**.49**	**.39**	**.000**
Positivity cost beliefs (T1)	.03	.05	−.06	.11	.03	.569
Value happiness beliefs (T1)	.06	.04	−.03	.14	.08	.181
Depressive symptoms (T1)	**−.19**	**.08**	**−.34**	**−.04**	**−.16**	**.013**
	DV = T2 Parental enhancing responses (*R* ^*2*^ = .23)					
Enhancing responses (T1)	**.42**	**.06**	**.31**	**.53**	**.45**	**.000**
Positivity cost beliefs (T1)	−.08	.07	−.21	.06	−.07	.288
Value happiness beliefs (T1)	.09	.06	−.03	.21	.10	.135
Depressive symptoms (T1)	−.04	.11	−.25	.16	−.03	.680
	DV = T2 Parental dampening responses (*R* ^*2*^ = .25)					
Parent education	**−.11**	**.05**	**−.20**	**−.02**	**−.14**	**.019**
Dampening responses (T1)	**.20**	**.07**	**.06**	**.33**	**.20**	**.004**
Positivity cost beliefs (T1)	.06	.07	−.09	.20	.05	.449
Value happiness beliefs (T1)	−.07	.06	−.19	.05	−.07	.267
Depressive symptoms (T1)	**.58**	**.11**	**.37**	**.80**	**.35**	**.000**

*Note*: DV = Dependent variable. T1 = Time 1. T2 = Time 2. Standard errors, confidence intervals, and *p* values are for unstandardized estimates. **Boldface** results are significant at *p* < .05.

Overall, the model fit the data well (*χ* ^*2*^(12) = 17.44, *p* = .134; RMSEA(90%CI) = .04 (.00, .08); CFI = .98; SRMR = .02; Hu & Bentler, 1999). All path coefficients are displayed in Table 2. Consistent with bivariate results, each set of socialisation behaviours demonstrated significant stability over time in multivariate analyses. For family savouring, over and above T1 levels and covariates (for which only being a mother predicted higher reported savouring over time), parents reporting greater depressive symptoms reported less family savouring over time. Neither positivity cost beliefs nor valuing PA beliefs predicted change in family savouring.

Contrary to hypotheses, there were no significant predictors of changes in enhancing behaviours over time. This was despite several significant bivariate relations both within and across time between parental beliefs, depression and enhancing responses.

Consistent with predictions, parental depressive symptoms at T1 positively predicted dampening responses at T2 over and above T1 dampening (and the significant covariate of parental education). In contrast, however, neither parental cost of positivity beliefs nor valuing PA beliefs significantly predicted changes in dampening responses.

Finally, we tested parent gender as a moderator. Because 16 parents did not indicate their gender identity, these moderation analyses were conducted on a subsample of 265 parents. To test moderation, a multigroup approach was used. Using a chi‐square difference test, model fit was compared for a model with all parameters freely estimated amongst mothers and fathers to a model in which parameters were constrained across gender. If the constrained model fit significantly worse, “omnibus” moderation would be indicated and followed by systematically testing each parameter across groups to identify specific paths that differed by parent gender. For this model, parent gender did not moderate any relations (Δ*χ* ^*2*^(13) = 17.01, *p* = .199).

## DISCUSSION

In this investigation of parental influences contributing to parental PA socialisation in India, our data show bivariate concurrent and longitudinal associations between parents' beliefs endorsing the value and cost of PA, as well as depressive symptoms to PA socialisation. However, in multivariate path analyses, only parents' depressive symptoms contribute to their responses to their adolescents' PA across time.

### Parental PA‐related beliefs predicting parental PA socialisation

Consistent with our prediction, at the bivariate level at T1, parents who indicated that PA is harmful reported engaging in more dampening responses to adolescent PA, and parents who reported higher endorsement of PA being valuable reported engaging in more enhancing and savouring responses. However, when examining these associations through path analyses across time, beliefs about PA did not predict socializations behaviours.

These data suggest that both sets of parents' beliefs (endorsing the value of PA, and its harmful effects) serve as correlates of parents' responses in expected directions; if parents consider PA to be harmful, they prefer to reduce PA experiences for their adolescent and respond with dampening, whereas if parents consider PA to be valuable, they prefer to maximise PA experiences for their adolescent. These findings replicated the cross‐sectional finding from a US‐based study demonstrating that parental cost of positivity beliefs were uniquely associated with dampening responses (Nyquist & Luebbe, 2021).

### Cultural considerations in beliefs and practices

Parents' PA‐related beliefs showed relatively low stability over time (correlations between T1 and T2 for cost of positivity and value of PA beliefs between .14 and .28, see Table 1), and there were positive associations between parental beliefs that PA is valuable and that PA is harmful. These associations suggest that parents likely hold both sets of beliefs that fit with the dialectical script of emotions that highlights a balance. According to this script, people in Asian cultures place an emphasis on balanced emotions as opposed to maximising PA (Miyamoto & Ryff, 2011), and our data (showing a positive rather than inverse association between views endorsing PA as valuable versus harmful) support this script.

The positive association may also indicate that parents consider PA as more acceptable in some situations versus others, and type and intensity of PA may be key (Parker et al., 2012). For example, parents may consider happiness as valuable, although they may consider expressions of pride in some situations as compromising social relations. Thus, type of PA (pride versus happiness) may be a critical determinant of parental PA‐related beliefs. In addition, we did not examine parental beliefs related to PA beyond whether it is considered viable or harmful. There may be additional dimensions of parental PA‐related beliefs that are important to examine. For example, parents may view PA within the context of controllability, changeability (e.g., developmental changes as children get older), and relational nature (e.g., its role in social connections; Parker et al., 2012).

Overall, low stability of parental PA related beliefs, positive associations between beliefs endorsing PA as harmful and PA as valuable, along with the finding that PA beliefs are associated with PA socialisation within time but not across time may indicate unique culturally salient processes. In communities where interdependence is valued, beliefs and behaviours are highly responsive to changing contexts. For example, parental beliefs about pride related to academic achievement may differ from their beliefs about happiness as a result of interacting with friends. Alternatively, there may be other cultural values that contribute to the variation in parents' PA beliefs over time, as well as to parental PA socialisation. A third possibility is that these findings may represent a universal process that parental beliefs endorsing the value and cost of happiness are a less effective predictor of parents' PA socialisation efforts.

### Parental depressive symptoms predicting parental PA socialisation

Consistent with the prediction, our findings demonstrated that at the bivariate level, parental depressive symptoms were positively associated with their dampening responses and negatively associated with family savouring. In addition, examining across time, parents with increased depressive symptoms at T1 engaged in less savouring and more dampening of PA with their adolescent at T2. These findings are consistent with research that suggests that parental depressive symptoms undermine the quality of parenting in general (Clark & Watson, 1991; Jaiswal et al., 2015), and that individuals who are depressed dampen their own (Clark & Watson, 1991) and their adolescents' PA (Nyquist & Luebbe, 2021). Taken together, these findings suggest that parents who experience depressive symptoms may not only experience a lack of PA themselves, they also struggle to facilitate their adolescents' PA. The current sample reported relatively average levels of depressive symptoms (mean ratings around 6), which are consistent with depressive symptomology reported in other community samples from India (Singh et al., 2013). These findings are noteworthy because even variability within the lower end of the depressive symptom spectrum seems to contribute to parents' responses to adolescents' PA. Future research that examines the association between parental depressive symptoms and their PA socialisation in clinic‐referred samples of parents may be helpful to understand the implications of moderate to high levels of parental depressive symptomatology.

Depressive symptoms showed moderate stability (*r* = .41). There were no significant bivariate associations between PA beliefs and depressive symptoms within or across time (except a weak positive association between parental cost of positivity belief and depressive symptoms at T1) suggesting that parents' depression did not contribute to low stability in their beliefs, and that parents' beliefs and mood are relatively independent of one another.

### Limitations and future directions

The current study was limited by the use of self‐report from a single respondent, potentially contributing to overestimation of effects due to shared method variance. Future studies may use observational methods to assess parental PA socialisation and/or include adolescent reports of parental responses. Because the current sample included middle‐class families from an urban city in India, the current findings may not be generalizable to parents in India from rural areas, low SES, or following religions not well represented in the current sample. There may be additional parent and adolescent variables that contribute to parental PA socialisation. For example, the context of the experience of PA may be relevant (e.g., academic success, relationship success), type of PA experienced (e.g., happiness versus pride), and how adolescents respond to parental socialisation. Future research may examine how these additional factors impact parental PA socialisation processes over time across a longer duration. Future research may also use open‐ended qualitative methods to explore parents' PA socialisation behaviours, and use these data to modify existing measures or develop new measures.

### Conclusion

The current study extends previous cross‐sectional work conducted in the USA to demonstrate that parental cognitions and mood are important correlates of parent behaviours in communities around the world. Furthermore, this study is the first to demonstrate predictive value of parents' depressive symptoms for their PA socialisation over time using a longitudinal design. These findings contribute to expanding theoretical frameworks of PA socialisation in diverse families, and have implications for parenting interventions that may target parental beliefs and mood to change parent behaviours.
